# Diagnostic value of tumour markers in pleural effusions

**DOI:** 10.11613/BM.2018.010706

**Published:** 2018-01-10

**Authors:** Darian Volarić, Veljko Flego, Gordana Žauhar, Ljiljana Bulat-Kardum

**Affiliations:** 1 Division of Pulmonology, Clinic of Internal Medicine, Clinical Hospital Centre Rijeka, Rijeka, Croatia; 2 Department of Internal Medicine, Faculty of Medicine, University of Rijeka, Rijeka, Croatia; 3 Department of Medical Physics and Biophysics, Faculty of Medicine, University of Rijeka, Rijeka, Croatia; 4 Department of Physics, University of Rijeka, Rijeka, Croatia

**Keywords:** pleural effusion, CA-125 antigen, carcinoembryonic antigen, neuron-specific enolase, cytokeratin 19 fragment

## Abstract

**Introduction:**

We investigated whether tumour markers carcinoembryonic antigen (CEA), neuron-specific enolase (NSE), cancer antigen 125 (CA-125), and cytokeratin 19 fragment (CYFRA 21-1) in pleural effusions and serum can be used to distinguish pleural effusion aetiology.

**Materials and methods:**

During the first thoracentesis, we measured pleural fluid and serum tumour marker concentrations and calculated the pleural fluid/serum ratio for patients diagnosed with pleural effusion, using electrochemiluminescence immunoassays. Receiver operating characteristic (ROC) analysis was carried out and the Hanley and McNeil method was used to test the significance of the difference between the areas under ROC curves (AUCs). In order to detect which tumour marker best discriminates between malignant and non-malignant pleural effusions and to establish the predictive value of those markers, discriminant function analysis (DFA) and logistic regression analysis were utilized.

**Results:**

Serum tumour markers CYFRA 21-1 and NSE as well as pleural NSE were good predictors of pleural effusion malignancy and their combined model was found statistically significant (Chi-square = 28.415, P < 0.001). Respective ROC analysis showed significant discrimination value of the combination of these three markers (AUC = 0.79).

**Conclusions:**

Serum markers showed superiority to pleural fluid markers in determining pleural fluid aetiology. Serum CYFRA 21-1 and NSE concentrations as well as pleural fluid NSE values had the highest clinical value in differentiating between malignant and non-malignant pleural effusions. The combination of these three markers produced a significant model to resolve pleural effusion aetiology.

## Introduction

Pleural effusion is the accumulation of liquid in the pleural cavity and, in most cases, is considered a result of a systemic or intrathoracic process. This pathological entity is a frequent problem in pulmonology and, though its incidence varies with clinical background, 90% of all pleural effusions are attributed to congestive heart failure, malignant processes, and pneumonia (*1*).

The main issues regarding pleural effusions are the differentiation of exudates and transudates and the accurate determination of effusion aetiology (*i.e.* whether the pleural effusion is malignant or non-malignant). The differentiation of exudates and transudates requires the evaluation of various biochemical parameters and their comparison in pleural fluid and serum. When differentiating transudates from exudates using classical Light’s criteria, it is helpful to recognize the pathogenic mechanism resulting in the pleural effusion. Recognizing the correct pathogenic mechanism is also useful for the purpose of differential diagnosis (*2*).

When referring to the effusion aetiology, non-malignant pleural effusions are twice as common as malignant effusions and have diverse causes and manifestations, making them a diagnostic challenge (*3*). Evaluating an effusion’s potential malignancy is crucial for patient management and prognosis. Cytological examination is frequently used as a simple and non-invasive procedure to evaluate the malignancy of a pleural effusion, but more procedures are needed to confirm malignant pleural effusion aetiology in the case of a negative cytological examination. The methods used for this confirmation, such as thoracoscopy, are often hazardous and difficult to perform. In an attempt to help in the differentiation of pleural effusion aetiologies, many studies have tried to examine tumour markers as a possible alternative to invasive procedures (*4*). Various studies have investigated the value of tumour markers in evaluating cancer patients as screening, diagnostic, prognostic, or monitoring tools (*5*-*8*).

The objective of the present study was to determine whether tumour-related biomarkers in pleural effusions and serum can be used to distinguish malignant and non-malignant pleural effusions. Our hypothesis was that tumour markers are a worthy substitute of the presence of malignant cells in pleural fluid as malignancy markers. We investigated four tumour markers in Croatian patients: carcinoembryonic antigen (CEA), neuron-specific enolase (NSE), cancer antigen 125 (CA-125), and cytokeratin 19 fragment (CYFRA 21-1).

## Materials and methods

### Subjects

**Figure 1:**
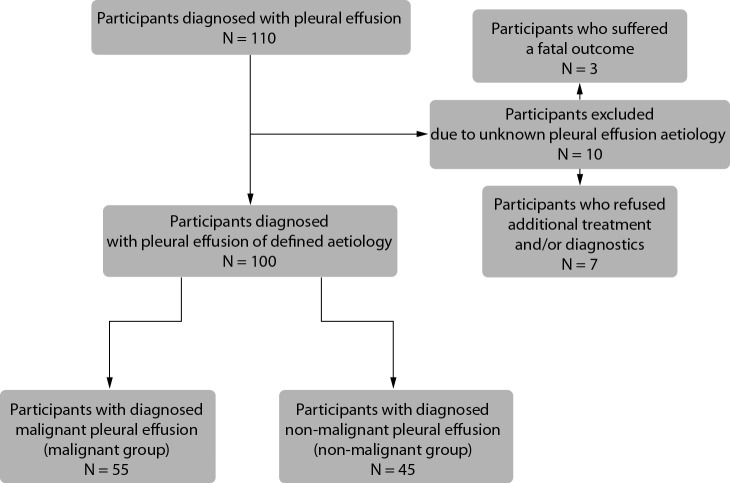
Flowchart showing the patient recruitment process

During the first thoracentesis, a total of 110 pleural fluid and serum samples were collected prospectively from 110 consecutive patients diagnosed with pleural effusion and admitted to the Department of Pulmonology at Clinical Hospital Centre in Rijeka, Croatia, between November 2013 and November 2014. Measurements were made at the time of admission to the Clinical Hospital Centre. After excluding 10 patients with unknown pleural effusion origin, 100 patients formed the study group. The 10 excluded patients were not followed up because 7 patients refused additional treatment and/or diagnostics and 3 suffered a fatal outcome (Figure 1).

Informed consent was obtained from all patients included in the study. All procedures were performed in accordance to the ethical standards of the institutional and national research committee, and the 1964 Helsinki declaration and its later amendments or comparable ethical standards. The study protocol was authorized by the ethics committee of the relevant Clinical Hospital Centre.

### Methods

Pleural effusion was diagnosed after thorough anamnesis, physical examination (lung percussion and auscultation), and chest x-ray. Pleural effusion was confirmed by thoracentesis.

Blood was sampled by venipuncture of antecubital veins. Pleural fluid was collected by a needle inserted between the seventh and ninth rib spaces, between the posterior axillary line and midline (thoracentesis). Both blood and pleural fluid were collected in 7 mL biochemical tubes without anticoagulants (BD vacutainer systems, Plymouth, UK). Blood and pleural specimens were centrifuged at a relative centrifugal force (RCF) of 1917xg for 10 minutes and the supernatant was analysed immediately. Blood specimens were tested at least 30 minutes after sampling, whereas pleural fluid samples were analysed immediately without previous storage. Tumour marker concentrations were determined by electrochemiluminescence immunoassays on the Cobas® e601 analyser (Roche-Diagnostics, Mannheim, Germany). The readers of the tests and reference standards were professionals, blind to the results of the other test.

Transudative and exudative pleural effusions were differentiated by Light’s criteria: effusion/serum protein ratio > 0.5, effusion/serum LD ratio > 0.6, effusion LD activity greater than two-thirds the upper limit of the laboratory’s reference range for serum LD (*9*, *10*). The finding of one of these criteria suggested exudate.

If malignant cells were detected in a smear of the pleural effusion sediment using the May-Grünwald-Giemsa staining method under a light microscope, the effusion was recognized as malignant. Pleural effusions from patients with previously diagnosed malignant illness were also considered malignant if other diseases were excluded as the cause. This method of diagnosing malignant pleural effusions was the usual clinical procedure used for defining malignant pleural effusions and regarded as the reference standard (*11*). Patients who had bacteria in the pleural effusion, severe febrile ailment, or lung infiltrates on chest x-ray with no other reason for pleural effusion were diagnosed with parapneumonic effusion or pyothorax (*12*). Patients who presented with physical symptoms of myocardial congestion and typical x-ray signs, also considering electrocardiography and echocardiography, were diagnosed with congestive heart failure. Other causes of pleural effusion, such as liver cirrhosis, chronic renal failure, and asbestosis, were also differentiated and classified as other known causes.

### Statistical analysis

Statistical analyses were performed using MedCalc software version 12.7.0.0 (Mariakerke, Belgium) and Statistica version 13 (Dell Inc., Tulsa, USA). Variables were tested for normality using a Kolmogorov-Smirnov test. Continuous variables with normal distribution were expressed as mean ± standard deviation (SD) and those with non-normal distribution as median and interquartile range (IQR). To test the difference in tumour marker concentrations between malignant and benign pleural effusions, we used the non-parametric Mann-Whitney U-test because the variables were not normally distributed. To determine the clinical usefulness of tumour markers in the studied group and assess the performance of tumour markers in distinguishing malignant and non-malignant pleural effusions, the marker values were reviewed by a receiver operating characteristic (ROC) analysis. The area under the ROC curve (AUC) serves as an overall measure of a biomarker/diagnostic test’s accuracy (*13*, *14*).

The diagnostic accuracy of each tumour marker and its pleural fluid/serum ratio was determined by sensitivity, specificity, positive predictive value (PPV), negative predictive value (NPV), positive likelihood ratio (LR+), negative likelihood ratio (LR-) and the AUC. We used Hanley and McNeil’s method for pairwise comparison of ROC curves in order to test the difference between the AUCs. P < 0.05 was considered significant.

In order to detect the tumour markers which best discriminate between malignant and non-malignant pleural effusions, discriminant function analysis (DFA) was employed. Logistic regression analysis was performed in order to establish the predictive value of those markers.

We calculated the required sample size using MedCalc software assuming that the desired statistical power of the test is 90% with a confidence level of 95% and the ratio of sample sizes in the negative and positive groups is 1. We wanted to show that the expected AUC of 0.7 for a particular test is significant from the null hypothesis value of 0.5. The required sample size was calculated to be 82. The present study had a slightly larger sample size to ensure that we would have enough results if some of the patients were excluded later.

## Results

The study group consisted of 73% males (73 male and 27 females). The median age of the study group was 71 years (range 22 - 92 years). In 88 patients (88%), we found a unilateral pleural effusion, whereas 12 (12%) patients were diagnosed with a bilateral effusion. The time interval between defining the pleural effusion as malignant or non-malignant and the determination of tumour marker concentrations was approximately 7 days. No adverse events occurred as a result of venipuncture or thoracentesis.

According to Light’s criteria, pleural exudates made up 71% (N = 71) of the study group; the other 29% (N = 29) were pleural transudates (Table 1). The main cause of pleural exudates was malignant disease while the main cause of pleural transudates was heart failure (Table 1).

**Table 1:** Differentiation of pleural effusions according to Light’s criteria with pleural fluid aetiology

**Pleural exudates**	**N / total**
malignant	55 / 71
parapneumonic	7 / 71
asbestosis	4 / 71
rib fracture	1 / 71
heart failure	1 / 71
liver cirrhosis	1 / 71
glomerulonephritis	1 / 71
hemopneumothorax	1 / 71
**Pleural transudates**	
heart failure	22 / 29
liver cirrhosis	3 / 29
chronic renal failure	2 / 29
asbestosis	1 / 29
rib fracture	1 / 29

Primary tumour sites in malignant pleural effusions were distributed as follows: lung (N = 17), pleura (N = 15), ovary (N = 4), colon (N = 4), stomach (N = 3), breast (N = 2), prostate (N = 2), malignant disease of unknown origin (N = 2), skin (N = 1), soft tissues (N = 1), pancreas (N = 1), oesophagus (N = 1), hypopharynx (N = 1), and lymph nodes (N = 1). The non-malignant or benign pleural effusion group (N = 45) consisted of all patients with pleural transudates or non-malignant pleural exudates, who were then compared to the patients diagnosed with malignant pleural effusions (N = 55). The median age of the patients with malignant pleural effusions was 70 (48 – 90) years; whereas the median age of the patients with non-malignant pleural effusions was 73 (22 – 92) years.

The gender distribution was 39 males and 16 females in the malignant group and 34 males and 11 women in the non-malignant pleural effusion group. The concentrations of tumour markers CEA, NSE, CA-125, and CYFRA 21-1 in pleural fluid (P), serum (S) and their pleural fluid/serum ratios (R) in the malignant and non-malignant patient group are presented in Table 2. Pleural fluid CEA, NSE and CYFRA 21-1, as well as serum NSE and CYFRA 21-1 concentrations were significantly higher in malignant effusions than in benign effusions. The pleural fluid/serum ratios for CEA and NSE were significantly higher in malignant than in benign effusions. CA-125 was the only marker to show no significant difference in pleural fluid or serum concentrations or their ratio.

**Table 2:** Tumour marker concentrations and pleural fluid/serum ratios in the malignant and non-malignant patient group

**Tumour marker**	**Malignant** **(N = 55)**	**Non-malignant** **(N = 45)**	**P**
(P) CA-125, mU/mL	814.3 (215.8 - 1107.0)	732.7 (278.1 - 984.6)	0.368
(S) CA-125, mU/mL	175.3 (42.5 - 299.5)	166.8 (62.2 - 303.5)	0.967
(R) CA-125	3.9 (1.9 - 8.2)	3.8 (1.7 - 7.5)	0.819
(P) CEA, µg/mL	3.0 (1.0 - 92.6)	0.9 (0.3 - 1.6)	< 0.001
(S) CEA, µg/mL	3.0 (1.6 - 6.7)	2.4 (1.3 - 3.6)	0.070
(R) CEA	0.9 (0.5 - 7.6)	0.4 (0.2 - 0.7)	< 0.001
(P) NSE, ng/mL	10.8 (5.3 - 31.0)	4.4 (2.2 - 11.2)	< 0.001
(S) NSE, ng/mL	19.4 (14.5 - 27.0)	12.7 (10.7 - 17.4)	< 0.001
(R) NSE	0.6 (0.3 - 1.3)	0.3 (0.2 - 0.1)	0.047
(P) CYFRA 21-1, ng/mL	57.5 (22.4 - 161.9)	13.0 (6.3 - 48.6)	< 0.001
(S) CYFRA 21-1, ng/mL	7.9 (3.6 - 14.9)	2.2 (1.4 - 4.4)	< 0.001
(R) CYFRA 21-1	7.7 (2.8 - 27.4)	4.0 (2.6 - 24.0)	0.484
Data are presented as median and interquartile range (IQR). P – pleural fluid concentration. S – serum concentration. R - pleural fluid/serum ratio. P < 0.05 was considered statistically significant.			

**Figure 2:**
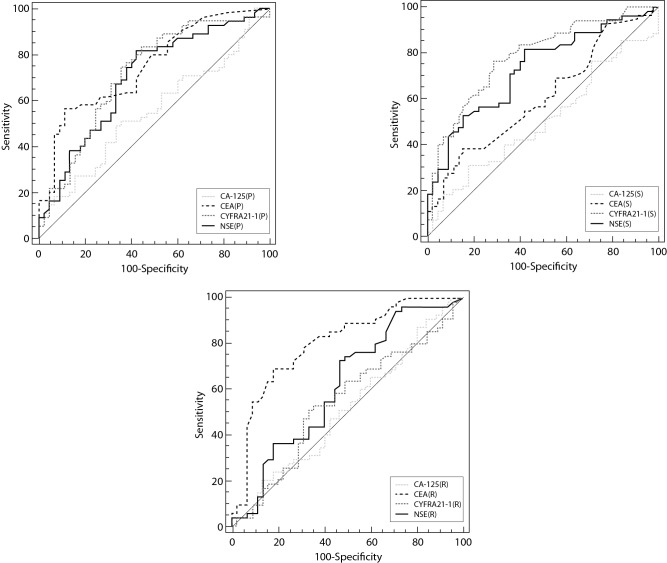
Receiver operating characteristics (ROC) curves for all parameters investigated in pleural fluid, serum and their ratios. P – pleural fluid concentration. S – serum concentration. R - pleural fluid/serum ratio.

The ROC analyses of the mentioned tumour markers (determined in pleural fluid, serum, and as pleural fluid/serum ratio) are shown in Table 3 with their cut-off points and corresponding diagnostic characteristics. We selected the positivity cut-off points after performing the test, choosing the ones that maximized tumour marker performance. The best cut-off values were based on the ROC analysis performed using MedCalc software. The marker values with the highest AUCs were the (R) CEA (AUC = 0.80), (S) CYFRA 21-1 (AUC = 0.79), (P) CEA (AUC = 0.75), (S) NSE (AUC = 0.73), (P) CYFRA 21-1 (AUC = 0.72) and (P) NSE (AUC = 0.70). The ROC curves for all measured entities are presented in Figure 2.

**Table 3:** Diagnostic accuracy characteristics for the investigated tumour markers and pleural fluid/serum ratios

**Tumour marker**	**Cut-off**	**Sensitivity**	**Specificity**	**PPV**	**NPV**	**AUC, P**	**LR+**	**LR-**
(P) CA-125, mU/mL	> 844.2	49.1 (35.4 - 62.9)	66.7 (51.0 - 80.0)	64.3 (48.0 - 78.4)	51.7 (38.1 - 65.2)	0.55 (0.45 – 0.65), 0.363	1.47	0.76
(S) CA-125, mU/mL	≤ 50.5	30.9 (19.1 - 44.8)	82.2 (67.9 - 92.0)	68.0 (46.5 - 85.1)	49.3 (37.6 - 61.1)	0.50 (0.40 – 0.60), 0.964	1.74	0.84
(R) CA-125	> 0.92	87.3 (75.5 - 94.7)	20.0 (9.6 - 34.6)	57.1 (45.9 - 67.9)	56.2 (29.9 - 80.2)	0.51 (0.41 – 0.62), 0.818	1.09	0.64
(P) CEA, µg/mL	> 2.2	56.4 (42.3 - 69.7)	88.9 (75.9 - 96.3)	86.1 (70.5 - 95.3)	62.5 (49.5 - 74.3)	0.75 (0.65 – 0.83), < 0.001	5.07	0.49
(S) CEA, µg/mL	> 3.9	38.2 (25.4 - 52.3)	84.4 (70.5 - 93.5)	75.0 (55.1 - 89.3)	52.8 (40.7 - 64.7)	0.61 (0.50 – 0.70), 0.061	2.45	0.73
(R) CEA	> 0.56	69.1 (55.2 - 80.9)	82.2 (67.9 - 92.0)	82.6 (68.6 - 92.2)	68.5 (54.3 - 80.6)	0.80 (0.71 – 0.874), < 0.001	3.89	0.38
(P) NSE, ng/mL	> 4.9	81.8 (69.1 - 90.9)	57.8 (42.2 - 72.3)	70.3 (57.6 - 81.1)	72.2 (54.5 - 86.0)	0.70 (0.60 - 0.79), < 0.001	1.94	0.31
(S) NSE, ng/mL	> 13.3	81.8 (69.1 - 90.9)	57.8 (42.2 - 72.3)	70.3 (57.6 - 81.1)	72.2 (54.5 - 86.0)	0.73 (0.63 – 0.82), < 0.001	1.94	0.31
(R) NSE	> 0.32	72.7 (59.0 - 83.9)	53.3 (37.9 - 68.3)	65.6 (52.3 - 77.3)	61.5 (44.6 - 76.6)	0.62 (0.52 – 0.71), 0.043	1.56	0.51
(P) CYFRA 21-1, ng/mL	> 14.1	83.6 (71.2 - 92.2)	55.6 (40.0 - 70.4)	69.7 (57.1 - 80.4)	73.5 (55.6 - 87.1)	0.72 (0.62 – 0.80), < 0.001	1.88	0.29
(S) CYFRA 21-1, ng/mL	> 3.5	76.4 (63.0 - 86.8)	71.1 (55.7 - 83.6)	76.4 (63.0 - 86.8)	71.1 (55.7 - 83.6)	0.79 (0.69 – 0.86), < 0.001	2.64	0.33
(R) CYFRA 21-1	> 7.62	50.9 (37.1 - 64.6)	66.7 (51.0 - 80.0)	65.1 (49.1 - 79.0)	52.6 (39.0 - 66.0)	0.54 (0.44 – 0.64) 0.489	1.53	0.74
P – pleural fluid concentration. S – serum concentration. R - pleural fluid/serum ratio. Sensitivity, specificity, positive predictive value (PPV), negative predictive value (NPV) and area under the curve (AUC) are presented as percentage with corresponding 95% confidence intervals. (LR+) - positive likelihood ratio. (LR-) - negative likelihood ratio. P < 0.05 was considered statistically significant.								

After pairwise comparison of all 12 ROC curves, significant differences were shown between AUCs of (S) CYFRA 21-1, (R) CEA, (P) CEA, (S) NSE, (P) NSE, (P) CYFRA 21-1, while no difference was found comparing AUCs of (P) CA-125, (R) CA-125, (S) CA-125 and (R) CYFRA 21-1 (data not shown). These results show that there are significant differences between diagnostic values of these tumour markers. However, there was no statistically significant difference between AUCs for tumour markers (S) CYFRA 21-1, (R) CEA, (P) CEA, (S) NSE, (P) NSE, (P) CYFRA 21-1 (data not shown) and therefore it cannot be concluded that any of them had greater diagnostic accuracy in distinguishing malignant from non-malignant pleural effusions. In order to better explain these results, DFA was performed.

Sets of four tumour markers concentrations measured in pleural fluid and serum, as well as their ratios were analysed with respect to their discriminatory ability, and the results presented in Table 4. Serum tumour markers have significant discriminant ability with respect to malignancy of pleural effusions, while pleural markers did not produce a significant model. However, individual marker’s analysis shows that one pleural fluid tumour marker (*i.e.* NSE) and two serum markers (*i.e.* NSE and CYFRA 21-1) discriminated well between groups with malignant and non-malignant pleural effusions. In order to establish the predictive value of those markers for classification with respect to malignancy of pleural effusion, logistic regression analysis was performed. The results presented in Table 5 confirm (S) CYFRA 21-1, (S) NSE and (P) NSE as good predictors of pleural fluid malignancy and establish the model of their combination as highly significant (P < 0.001). Additionally, respective ROC analysis shows good and significant discrimination value of (S) CYFRA 21-1, (S) NSE and (P) NSE combination.

**Table 4:** Discriminant function analysis parameters for discrimination between malignant and non-malignant pleural effusions

**Tumour marker**	**Wilks’ lambda**	**F**	**P**	**F (model)**	**P (model)**
(P) CA-125 (P) CEA (P) NSE (P) CYFRA 21-1	0.918 0.911 0.965 0.913	1.136 0.441 6.098 0.647	0.289 0.508 0.015 0.423	2.431	0.053
(S) CA-125 (S) CEA (S) NSE (S) CYFRA 21-1	0.854 0.853 0.903 0.907	0.254 0.101 5.693 6.136	0.616 0.751 0.019 0.015	4.139	0.004
(R) CA-125 (R) CEA (R) NSE (R) CYFRA 21-1	0.952 0.984 0.970 0.963	0.434 3.660 2.231 1.593	0.512 0.059 0.139 0.210	1.318	0.269
P – pleural fluid concentration. S – serum concentration. R - pleural fluid/serum ratio. P < 0.05 was considered statistically significant. Wilks' lambda test is used to test which variables significantly contribute in discriminant function analysis. F-value is associated with the respective partial Wilks' lambda. P-value is associated with the respective F-value.					

**Table 5:** Logistic regression parameters for prediction of malignant or non-malignant pleural effusions by individual markers and their combination

**Tumour marker**	**b**	**SE (b)**	**Wald**	**P**	**Tumour marker combination**			
**Chi-square**	**P**	**AUC**	**95% CI**
(S) CYFRA 21-1	0.096	0.041	5.507	0.019	28.415	< 0.001	0.79	0.69 - 0.86
(S) NSE	0.122	0.037	10.976	< 0.001				
(P) NSE	0.025	0.012	4.544	0.033				
P – pleural fluid concentration. S – serum concentration. R - pleural fluid/serum ratio. 95% CI – 95% confidence intervals. P < 0.05 was considered statistically significant. b - logistic regression coefficient and its standard error SE (b). The statistical significance of individual regression coefficients is tested using the Wald Chi-square statistic. If P < 0.05 then the variable contributes significantly to the outcome prediction. AUC – area under the ROC curve.								

Table 6 represents an aggregate contingency table for these three markers in relation to the diagnosis of malignancy. Using this table it is possible to determine the specificity and sensitivity of these tumour markers (Table 3), using MedCalc software.

**Table 6:** Serum tumour markers in relation to malignant and non-malignant effusions – contingency tables

**Tumour marker**	**Pleural effusions**		
**Malignant, N**	**Non-malignant, N**	**Total, N**
**(S) CYFRA 21-1**			
Positive (> 3.5 µg/mL)	42	14	56
Negative (≤ 3.5 µg/mL)	13	31	44
Total	55	45	100
**(S) NSE**			
Positive (> 13.3 ng/mL)	45	19	64
Negative (≤ 13.3 ng/mL)	10	26	36
Total	55	45	100
**(P) NSE**			
Positive (> 4.9 ng/mL)	45	19	64
Negative (≤ 4.9 ng/mL)	10	26	36
Total	55	45	100

## Discussion

Determining the diagnostic utility of tumour markers in pleural effusions, especially CEA, NSE, CA-125, and CYFRA 21-1, has many advantages with regard to both cost and the non-invasive approach, which is why we dedicated the entirety of this study to the analysis of these four markers. Targeted analysis of tumour markers in pleural effusions is acceptable, as the price of the test is not high. A cytomorphological finding of malignant cells in pleural effusions and immunocytochemical analysis can be used as alternative tests for greater diagnostic accuracy. Although these tumour markers exhibit great specificity, the low sensitivity of each individual marker limits their diagnostic value, and a few studies suggested they be used along with other pleural effusion tests for greater diagnostic accuracy (*15*). In the present study, serum markers were superior to pleural fluid markers in determining pleural fluid aetiology. Serum CYFRA 21-1, (S) NSE and (P) NSE were shown to be most significant predictors of pleural effusion malignancy and their combination produced a significant model for distinguishing malignant from non-malignant pleural effusions.

The value of a tumour marker lies in its ability to exclude or confirm the diagnosis of malignancy. In the first case, the marker with greatest sensitivity, NPV, and LR- should be used; in the second case (to confirm the diagnosis), tumour markers with the highest specificity, PPV, and LR+ should be used. One marker can be great for confirming diagnosis but not so good or very bad at excluding the disease and *vice versa*. In this attempt to analyse the diagnostic accuracy of these four tumour markers, we focused on determining the largest AUC, which combines optimum sensitivity and specificity in the differentiation of malignant and non-malignant pleural effusions. As the pairwise comparison of ROC curves yielded a large number of results, discriminant function analysis along with logistic regression analysis were performed.

Studies of the diagnostic value of tumour markers in pleural effusions are not rare, but they have not had definitive and clear results (*16*-*21*). One study of a Polish population concluded that CEA is the biomarker with the highest diagnostic utility in malignant pleural effusions, whereas CYFRA 21-1 has greater diagnostic value in serum than pleural fluid, and NSE has great sensitivity but low diagnostic value. It was concluded that pleural fluid markers were superior to serum markers in determining pleural fluid aetiology and that the combination of tumour markers may help improve their diagnostic accuracy (*16*). Our study confirmed that serum CYFRA 21-1 discriminates well between groups with malignant and non-malignant pleural effusions but on the other hand, CEA marker did not have the highest diagnostic utility and NSE was one of the best discriminators considering both serum and pleural values. Serum markers were found to be superior to pleural fluid markers in determining pleural fluid aetiology and the combination of (S) CYFRA 21-1, (S) NSE and (P) NSE produced a significant model for classification of malignant cases. Additionally, respective ROC analysis showed good and significant discrimination value of their combination (AUC = 0.79). In contrast, a study conducted in the Iranian population reported that the highest specificity was obtained with a combination of serum and pleural fluid CA 15-3 and NSE (100%), and a combination of serum and pleural fluid CA 15-3, NSE, and CEA (100%), whereas the highest sensitivity was obtained with a combination of serum and pleural fluid CA 15-3 and CEA (80%), as well as a combination of serum and pleural fluid CA 15-3, NSE, and CEA (80%) (*17*).

The median pleural fluid concentrations for the investigated tumour markers were higher in malignant effusions than benign effusions, with the exclusion of CA-125. This can be explained by the fact that pleural effusions are a filtrate of blood plasma and, therefore, the values of tumour markers do not significantly differ between pleural effusions and serum. Mechanisms involved in the production of pleural effusions are numerous, the most common of which are increased hydrostatic pressure in lung capillaries, reduction of colloid-osmotic blood pressure, increased capillary permeability in the lungs, decreased pressure in the pleural space, lymphatic vessel obstruction in the thorax, and increased pressures in the systemic vein bloodstream. The values of individual tumour markers depend on the dominating mechanism involved in the development of the pleural effusion. Pleural fluid CA-125 concentrations deviate from the other three markers tested in this study because CA-125 is also elevated in the serum of patients suffering from non-malignant diseases (*e.g.,* endometriosis, tuberculosis, purulent pleurisy and peritonitis, hepatitis, liver cirrhosis) (*18*). An examination of serum marker values showed that CYFRA 21-1 and NSE concentrations were higher in the group of patients diagnosed with malignant effusions, whereas there was no difference in CA-125 and CEA concentrations between the groups. This finding may be due to the fact that CYFRA 21-1 and NSE are more specific markers than CEA and CA-125. The pleural fluid/serum ratios of CEA and NSE were also higher in malignant effusions, whereas those of CA-125 and CYFRA 21-1 exhibited no differences.

The pleural fluid/serum ratio for CEA had one of the highest AUC values (*i.e*. 0.80) (*19*). The Polish study included the CEA marker ratio and reported an AUC of 0.84 (*16*). CEA concentrations in pleural fluid had the third highest AUC of 0.75. Pleural fluid CEA concentrations were reported in previous studies to have an AUC of 0.83, 0.72, and 0.86 (*16*, *20*, *21*). The dissimilarities between the results may be due to different primary diagnoses of the patients in the studies. When referring to serum CEA concentrations in our study, the AUC was 0.61. This marker has great specificity but lacks sensitivity, and when analysing the serum CEA AUC values in accordance with other examined markers, we found that the serum CEA concentrations do not have great diagnostic value.

Another valuable diagnostic marker according to the present study is CYFRA 21-1, but primarily considering the serum values. The AUC for serum CYFRA 21-1 was 0.79 and was proven by discriminant function analysis to be one of the best discriminators regarding pleural fluid aetiology. Logistic regression analysis also discovered serum CYFRA 21-1 to be one of the three best predictors of pleural effusion malignancy, according to this study. In a previous study by Korczynski *et al.* the AUC for serum CYFRA 21-1 was 0.78, whereas Li *et al.* differentiated malignant from benign effusions with a sensitivity of 56%, specificity of 87%, and diagnostic accuracy of 0.71 (*16*, *22*). Although serum CYFRA 21-1 concentrations are used primarily for the detection of epidermoid lung carcinoma, some studies have proposed this marker as a method of stratifying risk in mesothelioma, oral squamous cell carcinoma, and colorectal cancer (*23*-*26*). In our study, the pleural fluid concentrations of this marker had an AUC of 0.72, similar to NSE in serum and pleural fluid. A slightly lower AUC of 0.69 for serum CYFRA 21-1 was reported in the Polish study (*16*). The pleural fluid/serum ratio for CYFRA 21-1 was one of the worst diagnostic predictors, with an AUC of 0.54.

NSE is currently the most reliable tumour marker in the diagnosis, prognosis, and follow-up of small cell lung cancer (SCLC), even though increased NSE concentrations have been reported in non-small cell lung cancer (NSCLC) (*27*). We found an almost identical AUC value for NSE in serum and pleural fluid. The AUC for the serum measurements was 0.73, whereas the pleural measurements had an AUC of 0.70. The AUC for the NSE ratio was 0.62. Discriminant function analysis showed serum and pleural NSE values to be one of the best discriminators regarding pleural fluid aetiology and also logistic regression analysis proved these two markers to be two of the three best predictors with respect to malignancy of pleural effusion. According to this study, serum and pleural fluid NSE concentrations were found to be a more useful diagnostic tool than in results reported by Korczynski *et al.* and Lee *et al*. (*16*, *21*).

Although CA-125 was discovered 30 years ago, the Food and Drug Administration (FDA) still recommends it to monitor the response to therapy in patients with epithelial ovarian cancer and to detect residual or recurrent disease in patients who have undergone first-line therapy and who would be considered for second-look procedures (*28*). The present study showed that CA-125 has the lowest diagnostic utility of all markers studied. The AUC for this marker in the serum was 0.50, nearly the same value as the marker in pleural fluid (AUC = 0.55) and the pleural fluid/serum ratio (AUC = 0.51). In contrast, the AUC values for CA-125 in the Polish study were 0.60, 0.64, and 0.54 for serum, pleural fluid, and the ratio, respectively.

The present study has some limitations. Although the patients were included as a consecutive series, the fact that the study was performed in the Department of Pulmonology has the consequence of exposure to more diseases related to lung pathology. In addition, the number of patients included in this study is representative of the size of the medical facility where the study was performed. Furthermore, the present study does not evaluate a larger number of different tumour markers (*e.g.,* CA 15-3, CA 19-9, PSA, *etc.*) or compare their diagnostic utility with more classical laboratory tests (*e.g.* LD, albumin *etc.*).

The advantage of this study is that it is a real-time study conducted in a Croatian population; differences exist in the expression of particular tumour markers in different races and populations due to genetic susceptibility.

In conclusion, we have shown that certain tumour markers have a higher diagnostic value than others in Croatian patients. Serum markers were superior to pleural fluid markers in determining pleural fluid aetiology, according to this study. Serum CYFRA 21-1 concentrations, serum and pleural NSE values were found to be most significant predictors of pleural effusion malignancy and the combination of these three entities produced a significant model for distinguishing malignant from non-malignant pleural effusions. Further research in other populations and larger study groups is needed to determine whether measuring tumour markers in pleural effusions is appropriate in general.
